# What Is Our Understanding of the Influence of Gut Microbiota on the Pathophysiology of Parkinson’s Disease?

**DOI:** 10.3389/fnins.2021.708587

**Published:** 2021-08-26

**Authors:** Amaryllis E. Hill, Richard Wade-Martins, Philip W. J. Burnet

**Affiliations:** ^1^Medical Sciences Division, University of Oxford, Oxford, United Kingdom; ^2^Department of Physiology, Anatomy and Genetics, University of Oxford, Oxford, United Kingdom; ^3^Department of Psychiatry, University of Oxford, Oxford, United Kingdom

**Keywords:** Parkinson’s, microbiota, microbiome, bacteria, gut, dysbiosis, neurodegenerative, gut–brain

## Abstract

Microbiota have increasingly become implicated in predisposition to human diseases, including neurodegenerative disorders such as Parkinson’s disease (PD). Traditionally, a central nervous system (CNS)-centric approach to understanding PD has predominated; however, an association of the gut with PD has existed since Parkinson himself reported the disease. The gut–brain axis refers to the bidirectional communication between the gastrointestinal tract (GIT) and the brain. Gut microbiota dysbiosis, reported in PD patients, may extend this to a microbiota–gut–brain axis. To date, mainly the bacteriome has been investigated. The change in abundance of bacterial products which accompanies dysbiosis is hypothesised to influence PD pathophysiology *via* multiple mechanisms which broadly centre on inflammation, a cause of alpha-synuclein (a-syn) misfolding. Two main routes are hypothesised by which gut microbiota can influence PD pathophysiology, the neural and humoral routes. The neural route involves a-syn misfolding peripherally in the enteric nerves which can then be transported to the brain *via* the vagus nerve. The humoral route involves transportation of bacterial products and proinflammatory cytokines from the gut *via* the circulation which can cause central a-syn misfolding by inducing neuroinflammation. This article will assess whether the current literature supports gut bacteria influencing PD pathophysiology *via* both routes.

## Introduction

Parkinson’s disease (PD) is the second most common neurodegenerative disorder worldwide. Insoluble intracytoplasmic protein aggregates, primarily consisting of misfolded fibrillar alpha-synuclein (a-syn), are PD neuropathological hallmarks. These aggregates [Lewy bodies (LBs) and Lewy neurites (LNs)], are believed to cause the catecholaminergic (CA) and dopaminergic (DA) neuronal loss which manifests as motor dysfunction (parkinsonism). Since PD motor symptoms are not evident until approximately 60–70% of DA neurons in the substantia nigra pars compacta (SNpc) are lost (Dauer and Przedborski, 2003), the gut–brain hypothesis may allow earlier interventions to be made before the motor system becomes affected.

The microorganisms in the gut are involved in gastrointestinal (GI) homoeostasis, such as maintaining the integrity of the gut epithelial barrier (Miraglia and Colla, 2019), and their abnormal colonisation and function (dysbiosis) can lead to peripheral and/or systemic inflammation (Chen et al., 2019) which may facilitate PD pathophysiology by neural and humoral routes.

## Evidence for Gut Involvement in Parkinson’s Disease

Gastrointestinal symptoms have been shown to precede motor symptoms by Parkinson himself (Parkinson, 2002). Non-motor symptoms are thought to predate motor symptoms by approximately a decade and increase the risk of developing PD (Abbott et al., 2001; Adams-Carr et al., 2016). Prolonged colonic transit time is present in up to 80% of PD patients (Jost, 1997) with significantly higher constipation incidence also reported compared to healthy controls (HCs) (Edwards et al., 1991; Chen et al., 2015). Gut microbiota have been implicated since they can aid in host nutrient metabolism and modulate gastrointestinal motility (Miraglia and Colla, 2019).

### Pathophysiological Evidence

The characteristic Lewy bodies (LBs) have also been observed in the enteric nervous system (ENS) of PD patients (Wakabayashi et al., 1988; Shannon et al., 2012b; Gold et al., 2013). Braak et al. (2003) defined the caudo-rostral axis in the brain along which a-syn pathology progresses and identified LBs and LNs in the dorsal motor nucleus of the vagus (DMV) in PD post mortem brain samples. This implicated the vagus nerve, and later the ENS (Braak et al., 2006), in the spreading of PD pathology. Braak’s hypothesis states that retrograde transport of misfolded a-syn occurs from projection neurons in the ENS to the central nervous system (CNS), *via* the vagus nerve. The initial a-syn misfolding was posited to be induced in the enteric nerves by an exogenous, neurotropic pathogen (Lionnet et al., 2018). Indeed, other studies have corroborated this hypothesis (Shannon et al., 2012a; Stokholm et al., 2016).

## Neural and Humoral Routes for A-Synuclein Misfolding and Aggregation

Dysbiosis may explain the association between PD and risk factors known to influence microbiome composition, such as pesticides and diet (Gorecki et al., 2019; Gubert et al., 2020). Studies in PD, discussed below, propose that changes in bacterial products accompanying dysbiosis could contribute toward the observed inflammation in PD patients (Devos et al., 2013; Chen et al., 2019). Indeed, functional increases in intestinal epithelial barrier permeability (Davies et al., 1996; Kelly et al., 2014) and decreased expression of intestinal barrier tight junctions (Edelblum and Turner, 2009; Clairembault et al., 2015; Perez-Pardo et al., 2019) in PD have been attributed to inflammation. This increased permeability would allow translocation of bacteria and their products into the lamina propria, triggering further inflammation. Both neural and humoral routes converge on inflammation which, *via* oxidative stress, forms one way in which a-syn misfolding can occur (Hashimoto et al., 1999; Lema Tomé et al., 2013). Local inflammation facilitates peripheral a-syn misfolding which propagates to the brain *via* the neural route. Systemic inflammation induces neuroinflammation in the brain (Mogi et al., 1994) *via* the humoral route which causes a-syn misfolding. A-syn can induce further oxidative stress, forming a positive feedback loop which leads to neurodegeneration in the brain (Dias et al., 2013; Chen et al., 2019).

### Neural Route

Braak’s hypothesis forms the basis of the neural route. Dysbiosis in the gut lumen leads to a-syn aggregation in enteric nerves. Indirectly, inflammation increases intestinal barrier permeability and hence mucosal inflammation, from translocation of bacteria and their products, which facilitates a-syn misfolding (Forsyth et al., 2011; Lema Tomé et al., 2013; Kelly et al., 2014). Directly, enteroendocrine cells (EECs) may propagate misfolded a-syn from the lumen, along their neuropods, to enteric nerves *via* functional synapses (Chandra et al., 2017). This may explain how Braak’s luminal exogenous pathogen could directly cause a-syn aggregation in the enteric nerves without violating the gut epithelium. Peripheral misfolded a-syn is proposed to be transported in a prion-like way between neurons, passing from the enteric nerves to the vagus nerve to the brain, where a-syn forms intracytoplasmic aggregates (Visanji et al., 2013). Exogenous a-syn fibrils enter neurons (Volpicelli-Daley et al., 2011), seeding intracellular a-syn aggregation both *in vitro* (Luk et al., 2009) and *in vivo* (Kordower et al., 2011; Holmqvist et al., 2014; Okuzumi et al., 2018), and upon exiting these neurons restarts the process (Lee et al., 2005).

Various animal models have supported Braak’s hypothesis. Vagus nerve-mediated translocation of a-syn aggregations from the gut to the brain was observed after injections of human recombinant a-syn from PD patients into rats’ intestinal walls (Holmqvist et al., 2014), or the peritoneal cavity of a-synuclein overexpressing (ASO) mice (Breid et al., 2016). Furthermore, injection of preformed fibrils (PFFs) into mouse gastrointestinal tracts (GITs) led to DA neuronal loss in the SNpc and motor symptoms after 7 months, with a-syn accumulation in anatomical locations associated with more advanced Braak’s stages (Kim et al., 2019). In this study, truncal vagotomy in the PFF-injected mice prevented a-syn spreading to the brain and protected against loss of DA neurons. Decreased risk of PD with truncal vagotomy has also been observed in patients (Svensson et al., 2015; Liu et al., 2017).

### Humoral Route: Dysbiosis and Inflammation

Bacterial products and proinflammatory cytokines in the systemic circulation trigger neuroinflammation, inducing central a-syn misfolding through oxidative stress (Hashimoto et al., 1999; Lema Tomé et al., 2013). Although mechanistically distinct to the neural route, the direction of pathology transfer remains the same. The first study to link dysbiosis with PD demonstrated significant differences in faecal bacterial taxa between PD patients and HCs (Scheperjans et al., 2015). Many more case–control studies, using faecal samples, have followed (Table 1). Although heterogeneity between results exists, the consensus is that the dysbiosis observed represents a shift toward a proinflammatory profile.

**TABLE 1:** Table presenting results of statistically significant (*p* < 0.05) changes in abundance of bacterial taxa in faecal samples between Parkinson’s disease patients (PD) and healthy controls (HCs).

Studys	Increased in PD patient faeces	Decreased in PD patient faeces
Scheperjans et al. (2015)	Family: Lactobacillaceae, Verrucomicrobiaceae, Bradyrhizobiaceae, Ruminococcaceae	Family: Prevotellaceae, Clostridiales i.s. IV
Keshavarzian et al. (2015)	Phylum: Bacteroidetes, Proteobacteria, Verrucomicrobia Family: Bacteroidaceae, Clostridiaceae, Verrucomicrobiaceae Genus: *Akkermansia*, *Oscillospira*, *Bacteroides*	Phylum: Firmicutes Family: Lachnospiraceae, Coprobacillaceae Genus: *Blautia*, *Coprococcus*, *Dorea*, *Roseburia*
Hasegawa et al. (2015)	Genus: *Lactobacillus*	Species: *Bacteroides fragilis*, *Clostridium coccoides*, *Clostridium leptum*
Unger et al. (2016)	Family: Enterobacteriaceae Genus: *Bifidobacterium*	Phylum: Bacteroidetes Family: Lactobacillaceae, Enterococcaceae Species: *Faecalibacterium prausnitzii*
Bedarf et al. (2017)	Phylum: Firmicutes, Verrucomicrobiaceae Genus: Unclassified *Firmicutes*, *Akkermansia*	Family: Erysipelotrichaceae, Prevotellaceae Genus: *Eubacterium*, *Prevotella*
Hill-Burns et al. (2017)	Family: Bifidobacteriaceae, Christensenellaceae, Lactobacillaceae, Tissierellaceae, Verrucomicrobiaceae Genus: *Akkermansia*, *Lactobacillus*, *Bifidobacterium*	Family: Lachnospiraceae, Pasteurellaceae
Hopfner et al. (2017)	Family: Lactobacillaceae*, Barnesiellaceae, Enterococcaceae	n/a
Petrov et al. (2017)	Genus: *Bifidobacterium*, *Catabacter*, *Christensenella*, *Lactobacillus*, *Oscillospira*	Genus: *Bacteroides*, *Dorea*, *Faecalibacterium*, *Prevotella*
Li et al. (2017)	Phylum: Proteobacteria, Actinobacteria Family: Enterobacteriaceae, Veillonellaceae, Erysipelotrichaceae, Coriobacteriaceae, Streptococcaceae, Moraxellaceae, and Enterococcaceae Genus: *Acidaminococcus*, *Acinetobacter*, *Enterococcus*, *Escherichia–Shigella*, *Megamonas*, *Megasphaera*, *Proteus*, *Streptococcus*	Phylum: Bacteroidetes Genus: *Blautia*, *Faecalibacterium*, *Ruminococcus*
Heintz-Buschart et al. (2018)	Phylum: Verrucomicrobia Class: Verrucomicrobiae Order: Verrucomicrobiales Genus: *Akkermansia*	n/a
Lin et al. (2018)	Family: Eubacteriaceae, Bifidobacteriaceae, Aerococcaceae, Desulfovibrionaceae	Phylum: Firmicutes, Tenericutes, Euryarchaeota Family: Streptococcaceae, Methylobacteriaceae, Comamonadaceae, Halomonadaceae, Hyphomonadaceae, Brucellaceae, Xanthomonadaceae, Lachnospiraceae, Actinomycetaceae, Sphingomonadaceae, Pasteurellaceae, Micrococcaceae, Intrasporangiaceae, Methanobacteriaceae, Idiomarinaceae, Brevibacteriaceae, Gemellaceae
Qian et al. (2018)	Genus: *Clostridium IV*, *Sphingomonas*, *Holdemania*, *Clostridium XVIII*, *Butyricicoccus*, *Anaerotruncus*, *Aquabacterium*	n/a
Barichella et al. (2019)	Phylum: Proteobacteria, Verrucomicrobia Family: Enterobacteriaceae, Verrucomicrobiaceae, Bifidobacteriaceae, Christensenellaceae, Coriobacteriaceae, Lactobacillaceae Genus: *Akkermansia*	Family: Lachnospiraceae
Li et al. (2019)	Family: Ruminococcaceae, Verrucomicrobiaceae, Porphyromonadaceae, Hydrogenoanaerobacterium, Lachnospiraceae NK4A	Family: Bacteroides, Prevotellaceae
Pietrucci et al. (2019)	Family: Lactobacillaceae, Enterobacteriaceae, Enterococcaceae	Family: Lachnospiraceae
Vidal-Martinez et al. (2020)	Family: Verrucomicrobiaceae Genus: *Akkermansia*	n/a
Ren et al. (2020)	PD-MCI (mild cognitive impairment) Vs. PD-NC (normal cognition) and HC: Genus: *Blautia, Ruminococcus* PD-NC vs. PD-MCI and HC: Family: Rikenellaceae Genus: *Alistipes*, *Barnesiella*, *Butyricimonas*, *Odoribacter*	n/a
Zhang et al. (2020)	Phylum: Firmicutes, Actinobacteria, Verrucomicrobia Genus: *Oscillospira*, *Akkermansia*	Phylum: Bacteroidetes, Fusobacteria Genus: *Fusobacterium*

*Further statistical analysis rendered change non-statistically significant.

Short-chain fatty acids (SCFAs) are produced by GI bacteria when anaerobically fermenting dietary fibres. These SCFAs (in particular butyrate, propionate and acetate) have anti-inflammatory effects both locally and systemically (Millard et al., 2002; Dalile et al., 2019). Reductions in the butyrate-producing families Lachnospiraceae and Prevotellaceae and bacterial genera such as *Blautia*, *Roseburia*, *Coprococcus*, and *Faecalibacterium prausnitzii* are most commonly found (Table 1). Decreased faecal SCFA concentrations in PD have been documented (Unger et al., 2016), which could increase local inflammation and in turn peripheral a-syn misfolding, facilitating the neural route. Since SCFAs can maintain the integrity of the intestinal barrier (Wang et al., 2012), their reduction could increase gut barrier permeability, facilitating the passage of other bacterial products and proinflammatory cytokines into the circulation, thereby engaging the humoral system (Dalile et al., 2019). Moreover, in normal physiology unmetabolised SCFAs can reach the systemic circulation and cross the blood–brain barrier (BBB) (Mitchell et al., 2011), following the humoral route, meaning that a lack of SCFAs could directly contribute to neuroinflammation. However, reports of SCFA-producing bacteria abundance are contradictory: *Prevotella* is decreased across studies whilst *Akkermansia* is increased, despite both containing mucin-degrading species (Table 1). This same pattern has been identified in multiple sclerosis (Freedman et al., 2018). Decreased *Prevotella* levels may reflect a lack of mucin synthesis, linked to increased barrier permeability (Bullich et al., 2019). Indeed, butyrate stimulates mucin synthesis (Brown et al., 2011) and putative-butyrate-producing (pBP) bacteria, such as *F. prausnitzi* and *Roseburia*, are consistently decreased (Table 1). *Akkermansia* may function as a double-edged sword: although mucin degradation is pro-inflammatory, decreased mucin levels could negatively feedback and increase other bacteria’s mucin synthesis (Bullich et al., 2019). Moreover, *Akkermansia* converts mucin degradation products into SCFAs (Derrien et al., 2004). Therefore, without pBP bacteria decreases, increased *Akkermansia* could be anti-inflammatory; however, decreased pBP bacteria abundance could cause net mucin degradation and increased barrier permeability.

Molecular H2 is another bacterial fermentation product which could be affected by dysbiosis. H2 has anti-inflammatory and antioxidant properties (Ostojic, 2018). Reduced intestinal H2 production in PD, through decreases of *Clostridium* and *Prevotella* and species such as *Bacteroides fragilis* (Table 1), may compromise the function of tissues which use it (such as DA neurons) (Ostojic, 2018). Indeed, motor symptoms in rodent lesion models of PD were prevented by H2S inhalation and systemic administration of NaHS (an H2S donor), and DA neuronal loss was reduced (Hu et al., 2010; Kida et al., 2011). Therefore, a decrease in H2 might predispose to DA neuronal loss and hence PD pathology *via* the humoral route.

The bacterial endotoxin, LPS, is also implicated in PD pathogenesis and may arise from the enrichment of Gram-negative-rich phyla such as Proteobacteria and Verrucomicrobia (Table 1). Increased TLR4 (LPS-specific receptor) expression in PD colonic biopsies (Perez-Pardo et al., 2019) and decreased serum LPS-binding protein (LBP) concentrations in PD (Forsyth et al., 2011; Hasegawa et al., 2015), also indicated LPS involvement. Functional evidence comes from rotenone-treated TLR4-KO mice which, compared to rotenone-treated WT mice, had reduced inflammation (intestinal and of the brain) and dysfunction (intestinal and motor) (Perez-Pardo et al., 2019). LPS can subvert the intestinal epithelial barrier both indirectly, through induced proinflammatory cytokines, and directly (Forsyth et al., 2011; Pawłowska and Sobieszczańska, 2017). LPS-induced inflammation in the lamina propria facilitates the neural route and, by entering the systemic circulation, LPS can directly participate in the humoral route. Moreover, LPS can disrupt the BBB (Kortekaas et al., 2005; Banks and Erickson, 2010), and in the brain can activate microglial CD14/TLR4/LBP complexes (Rivest, 2003). This creates a positive feedback cycle whereby microglia release proinflammatory cytokines, causing neuroinflammation which results in neuronal death and release of a-syn which then binds to TLR4 and/or TLR2 to further activate microglia and astroglia (Fellner et al., 2013; Kim et al., 2013). This process can mediate DA neuronal loss in the SN *via* oxidative stress (Qin et al., 2007; Dias et al., 2013).

Bacterial amyloids are increasingly being implicated in PD pathology *via* the neural route or exacerbating existing pathology *via* the humoral route. Extracellular amyloid fibres, such as curli, are produced by bacterial species including *Escherichia coli* (Römling et al., 1998; Hufnagel et al., 2013). Since bacterial amyloids can cross-seed amyloids from other bacterial species to induce aggregation, they may also cross-seed human a-syn in enteric nerves for propagation *via* the vagus nerve (Santos et al., 2019). Evidence for this comes from a study which exposed aged rats with human-a-syn-expressing-*Caenorhabditis elegans* to curliated *E. coli*: rats had increased a-syn inclusions in the gut and brain, accompanied by neuroinflammation (Chen et al., 2016). Furthermore, administration of curliated *E. coli* to ASO mice produced motor defects and GI dysfunction in addition to increasing gut and brain a-syn aggregation (Sampson et al., 2020).

A role for bacterial amyloids in PD would presumably require a significant increase in amyloid-synthesising bacteria. Although none of the studies summarised in Table 1 investigated amyloid-synthesising bacteria, consistent enrichment of *E. coli* is observed, though without changes in other amyloid-producing bacteria such as *Streptococcus mutans*, *Staphylococcus aureus*, and *Mycobacterium tuberculosis*. This potential discrepancy may be resolved by hypotheses that bacterial amyloids from different bacterial species induce cross-seeding in a strain-specific manner, i.e., solely on a-syn (Friedland and Chapman, 2017), meaning that even small quantities of bacterial amyloids could initiate pathology.

Bacterial amyloids and human oligomeric a-syn are recognised by the host immune system *via* the gut mucosal TLR2/TLR1 heterocomplex (Tükel et al., 2010; Nishimori et al., 2012; Hufnagel et al., 2013; Kim et al., 2013; Daniele et al., 2015). This would initiate a local and central immune response against endogenous a-syn (Lindestam Arlehamn et al., 2020), creating a proinflammatory environment which could facilitate a-syn misfolding (Hufnagel et al., 2013; Friedland and Chapman, 2017; Miraglia and Colla, 2019).

## How Useful Are These Studies?

Some common criticisms can be applied to these studies. Methodological differences, some of which are highlighted in Supplementary Table 1, may explain some of the heterogeneity between results. Many studies did not identify potential confounders in statistical analyses which may have resulted in false positive outcomes. For example, dietary differences between PD and HC groups, which could account for differential microbial composition (Graf et al., 2015), were not assessed. Sample handling methods (not listed), such as the time period between collection and freezing of samples, also varied greatly but were not considered as potential confounders (Haikal et al., 2019). Future studies need to agree on a standardised protocol, with more stringent inclusion/exclusion criteria, to increase the reproducibility and hence the reliability of the reported results.

## Cause or Consequence?

It is difficult to discern whether dysbiosis is a cause or consequence of PD. Although two longitudinal studies have been conducted in PD patients, neither have reported significant changes in microbial composition with progression (Minato et al., 2017; Aho et al., 2019). Support for a causative role comes from the association between PD risk and inflammatory conditions where dysbiosis is also reported, such as irritable bowel syndrome (IBS) and inflammatory bowel disease (IBD) (Lai et al., 2014; Lin et al., 2016; Mertsalmi et al., 2017). Evidence for the ENS controlling microbial composition, with the CNS modulating these ENS signals (Rolig et al., 2017), suggests that dysbiosis is a consequence of PD. This substantiates the hypothesis that loss of central DA neurons initiates DMV degeneration which results in GI inflammation and hence dysbiosis (Ulusoy et al., 2017; Rolli-Derkinderen et al., 2020). However, the decreased PD risk after vagotomy would suggest pathology originating from a region innervated by the vagus nerve, such as the GIT, refuting the CNS-centric hypothesis (Svensson et al., 2015; Liu et al., 2017; Kim et al., 2019). These opposing arguments could potentially be reconciled by an emerging theory which suggests the existence of different subtypes of PD: “PNS-first” or “CNS-first” (Borghammer and Van Den Berge, 2019). If a-syn pathology starts in the PNS in a subset of patients, early interventions to manipulate gut microbiota could be performed to halt the progression of neurodegeneration before motor system involvement. Faecal microbiota transplantation (FMT) studies in humans have produced varying results: whilst Xue et al. (2020) found improvements in clinical scoring scales (such as UPDRS and NMSS), Huang et al. (2019) did not but noted constipation alleviation. These studies’ sample sizes and follow-up periods are too small to draw conclusions from, however, a larger clinical trial is currently ongoing (Santens, 2021 – NCT03808389). Probiotics administration in PD patients produced significant improvements (i.e., decreases in MDS-UPDRS score) (Tamtaji et al., 2019) which has been supported by DA neuron neuroprotective effects and decreased motor impairment observed after probiotic administration in a PD mouse model (Hsieh et al., 2020).

Seminal evidence for the role of GIT microbiota in PD pathogenesis comes from a study of germ-free (GF) ASO mice by Sampson et al. (2016). These mice had reduced a-syn pathology load, microglial activation and motor symptoms compared to specific pathogen-free (SPF) ASO mice, implicating gut microbiota in causing PD pathophysiology. Moreover, GF-ASO mice transplanted with faecal microbiota from human PD donors developed exacerbated motor symptoms compared to those given HCs (Sampson et al., 2016). This translates the associative evidence from Table 1 into causal evidence.

## Summary of Gut Microbial Mechanisms Underlying PD Pathophysiology

**FIGURE 1:**
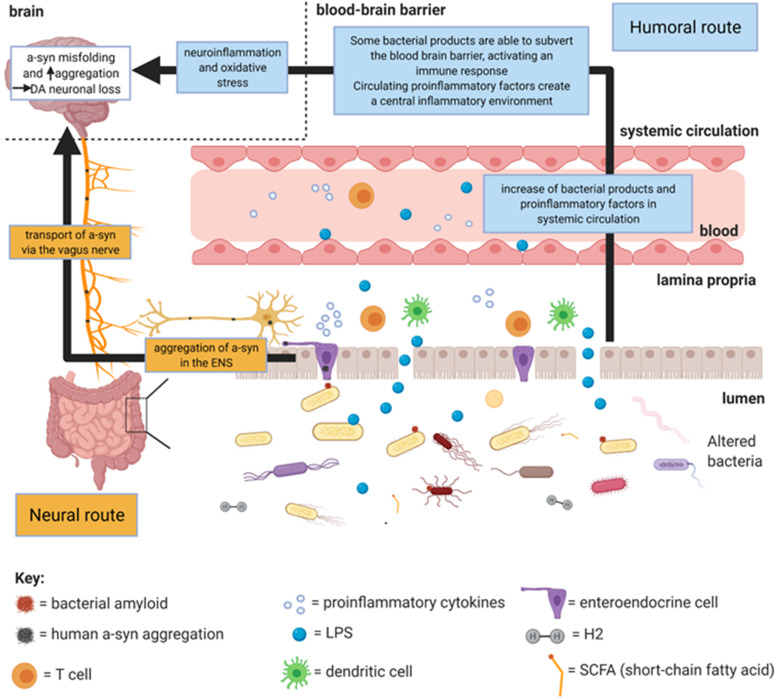
Summary diagram of the main mechanisms by which gut microbiota dysbiosis (specifically of bacteria) may cause PD pathophysiology. Created with BioRender.com.

The routes linking gut microbiota to PD pathophysiology are illustrated in Figure 1. The change in SCFA- and H2-producing bacteria, which accompanies dysbiosis, initiates a local proinflammatory environment which triggers a-syn misfolding peripherally in the gut, thus facilitating the neural route. Bacterial amyloids may also induce peripheral a-syn misfolding independently of inflammation. Peripheral misfolded a-syn can be transported along the enteric nerves *via* the vagus nerve to the brainstem. Once in the brain, a-syn progresses along Braak’s caudo-rostral axis. Local inflammation from dysbiosis can also increase the permeability of the gut brain barrier, allowing bacterial products to enter the lamina propria which can generate further inflammation. Some of these bacterial products (e.g., LPS) and proinflammatory cytokines can enter the circulation from the lamina propria, acting *via* the humoral route by generating neuroinflammation which causes a-syn misfolding.

## Conclusion

Microbiota undoubtedly play a role in PD pathophysiology. Associative evidence from case–control studies and functional evidence from animal models have provided support for microbiota causing PD *via* the neural and humoral routes. PD is primarily considered a disease of old age, despite pathogenesis preceding motor symptoms by years, with dysbiosis conceivably acting to exacerbate inflammation. Whilst previously gut dysbiosis was only considered as a consequence of PD, it is now also accepted that bacterial products may influence PD pathology through creating peripheral and systemic inflammatory environments, increasing both peripheral a-syn transport to the brain and neuroinflammation (Perez-Pardo et al., 2017). However, it is still not clear what initially triggers dysbiosis nor how inflammation would selectively cause a-syn aggregation rather than, for example, aggregation of Aβ in Alzheimer’s disease since inflammation is an underlying feature of many neurodegenerative diseases. Other factors are most likely implicated, such as gene mutations (e.g., in clearance mechanisms for misfolded a-syn), since not every ageing person develops PD or a neurodegenerative disease (Tran et al., 2020).

Understanding the exact mechanisms by which dysbiosis could lead to the neuropathological hallmarks of PD will require case–control studies to shift from predominantly bacterial abundance measures to whole metagenome sequencing (Supplementary Table 1) which provides data on functional changes in the microbiota as well as the levels of other microbes such as yeasts and viruses (Scheperjans, 2016). Studies which look set to strengthen evidence for gut bacteria involvement are investigating the effects of antibiotic administration on PD risk, since antibiotics alter gut bacteria composition (Mertsalmi et al., 2020).

## Author Contributions

AH and PB made an equal contribution to the conceptualisation, drafting of the manuscript, scientific content proof-reading. AH drafted the final version of the document and constructed the accompanying figure and table. RW-M provided a significant contribution to clinical content and proof-reading. All authors contributed to the article and approved the submitted version.

## Conflict of Interest

The authors declare that the research was conducted in the absence of any commercial or financial relationships that could be construed as a potential conflict of interest.

## Publisher’s Note

All claims expressed in this article are solely those of the authors and do not necessarily represent those of their affiliated organizations, or those of the publisher, the editors and the reviewers. Any product that may be evaluated in this article, or claim that may be made by its manufacturer, is not guaranteed or endorsed by the publisher.
