# Supplementary material for: What Is Our Understanding of the Influence of Gut Microbiota on the Pathophysiology of Parkinson’s Disease?
Source: Front Neurosci. 2021 Aug 26;15:708587. doi: 10.3389/fnins.2021.708587 (PMC8432298; doi:10.3389/fnins.2021.708587)
Supplement: Supplementary file 1 [file Table_1.DOCX]

Supplementary Material

**Table S1.** Methodological differences between the case-control papers from Table 1.

| **Study** | **Sequencing techniques** | **Sample size** | **Disease duration-years (mean) (SD)** | **Advantages** | **Drawbacks** |
| --- | --- | --- | --- | --- | --- |
| Scheperjans et al. 2015 | 16S rRNA  (V1-V3) | N= 144  PD: 72  HC: 72 | n/a | - Sex and age-matched (± 5 years). - Accounted for confounders in calculations. | - Dietary habits for subjects were not controlled for. |
| Keshavarzian et al. 2015 | 16S rRNA (V4) | N= 72  PD: 38  HC: 34 | 6.4 (4.7) | - No significant differences in sex and BMI. - Dietary data collected and no significant differences identified. | - 1/3 of PD subjects were drug-naïve. - Not age-matched. - Did not identify and assess confounders other than diet. |
| Hasegawa et al. 2015 | qRT-PCR of selected taxa | N= 88  PD: 52  HC: 36 | 9.5 (5.4) | - No significant differences in age or sex. | - For diet, only the frequency of subjects taking lactic acid bacteria-containing beverages was controlled. - Did not identify and assess confounders. - Only 19 taxa investigated. |
| Unger et al. 2016 | qRT-PCR of selected taxa | N= 68  PD: 34  HC: 34 | 6.8 | - Age-matched. - All PD patients on dopaminergic drugs. | - Authors argued that since all on omnivorous diets, dietary habits were unlikely to be a major confounder which is an oversight. - Did not assess other confounders between the two groups. - Only 8 taxa investigated. |
| Bedarf et al. 2017 | Shotgun metagenomics | N= 59  PD: 31  HC: 28 | <1 | - Age-matched. - All PD subjects L-DOPA naïve. - Functional analysis of metagenome. | - All men. - Sample size is small so unable to confidently identify confounders. |
| Hill-Burns et al. 2017 | 16S rRNA (V4) | N= 327  PD: 197  HC: 130 | 13.7 (6.5) | - Analysed confounders (including medication and diet) and took them into account in statistical analyses. - Large sample size. | - Some identified confounders could have been initially controlled for in the exclusion/ inclusion criteria (e.g age, sex). - Functional predictive analysis. |
| Hopfner et al. 2017 | 16S rRNA (V1-V2) | N= 58  PD: 29  HC: 29 | n/a | - Age-matched. - HCs chosen which had same direct environment (and hence a similar diet) to PD patients. - Controlled for confounders in an additional section and eliminated significance of *Lactobacillaceae.* - All PD subjects were taking anti-parkinsonian drugs. | - Sex-ratio differed - Fermented milk products seemed to be only dietary factor controlled. - Did not control for confounders in main results. - Small sample size. |
| Petrov et al. 2017 | 16S rRNA (V3-V4) | N= 155  PD: 89  HC: 66 | n/a | - No significant difference in age between the two groups. | - Data on subject sexes not reported. - Dietary data not collected. - No evidence of confounders being identified or accounted for. |
| Li et al. 2017 | 16S rRNA (V3-V5) | N= 38  PD: 24  HC: 14 | n/a | - Age and sex-matched. - No statistically significant difference in BMI. | - Small sample size. - Did not control for medication and diet of subjects. - No evidence of confounders being identified or accounted for. |
| Heintz-Buschart et al. 2018 | 16S rRNA (V4) | N= 154  PD: 76  HC: 78 | 6.0 (2.6) | - No statistically significant difference in mean age and sex ratio. - Identified potential confounders and analysed their significance. | - No data on diet. |
| Lin et al. 2018 | 16S rRNA (V4) | N= 120  PD: 75  HC: 45 | 4.5 | - Age-matched PD and HCs (HCs were the spouse of PD patients where possible to limit environmental and dietary variation). - No significant difference in sex ratio. - Analysed dietary habits and determined it had no effect on microbial composition. - Assessed potential confounders. - All PD subjects were taking anti-parkinsonian drugs. |  |
| Qian et al. 2018 | 16S rRNA (V3-V4) | N= 90  PD: 45  HC: 45 | 5.7 (4.1) | - Couples used (one PD patient and one HC) = share more of same gut microbiota and this minimises dietary and environmental variation. - No significant difference in age, sex or BMI. - Assessed significance of potential confounders using a GLM (general linear model). | - Small sample size. |
| Barichella et al. 2019 | 16S rRNA (V3-V4) | N= 306  PD: 193  HC: 113 | 7.7 (7.3) | - Large sample size. - Dietary data recorded. - Assessed multiple confounders. | - Functional predictive analysis. |
| Li et al. 2019 | 16S rRNA (V3-V4) | N= 20  PD: 10  HC: 10 | 6.2 (5.6) | - No significant difference in sex ratio, age, BMI, blood sugar and blood pressure. - Assessed significance of potential confounders. | - Small sample size. - Dietary habits not investigated. |
| Pietrucci et al. 2019 | 16S rRNA (V3-V4) | N= 152  PD: 80  HC: 72 | n/a | - Large sample size. - Aimed for HCs to be caregivers of PD partners to minimise dietary & environmental differences. - Thorough dietary habit history taking. - Confounding factors recognised and taken into account. | - Functional predictive analysis. |
| Vidal-Martinez et al. 2020 | 16S rRNA (V3-V4) | N=22  PD: 9  HC: 13 | n/a |  | - Small sample size. - Did not control for medication or diet of subjects. - No evidence of confounders being identified or accounted for. - Sex of participants and average age of each group not reported, suggesting that the groups were not age- and sex-matched. - Both PD and HCs were “recruited by flyers posted in clinics and hospitals” which casts doubt over how healthy the HCs were. |
| Ren et al. 2020 | 16S rRNA  (V3-V4) | N=40  PD-MCI: 13  PD-NC: 14  HC: 13 | PD-MCI: 7.00 (8.07)  PD-NC: 5.64 (3.34) | - Assessed differences in microbiota not only between HCs and PDs patients but also between PD patients with different cognitive abilities: MCI (mild cognitive impairment) and NC (normal cognition). - Identified potential confounders (including age, sex and antiparkinsonian medication) and accounted for them by using a GLM. - Healthy controls were spouses from PD patients (9 from PD-NC group and 4 from PD-MCI group), minimising environmental and dietary differences between patients and controls. | - Small sample size. - Functional predictive analysis. |
| Zhang et al. 2020 | 16S rRNA  (V4) | N=200  PD: 63  HC: 137 | 4.8 (2.2) | - No significant differences in age or sex. - 63 of the HCs were healthy spouses (HS) of the PD patients, minimising environmental and dietary differences. The remainder of the HCs were age-matched healthy people (HP). - Identified antiparkinsonian medication and environmental differences as potential confounders and analysed their effects on microbial composition. They determined that the antiparkinsonian medication had no significant effect on microbial composition. | - No dietary information was collected from the HP HCs. |
